# Testing the effectiveness of the culturally adapted skills training START NOW to reduce mental health problems in adolescent refugees: study protocol for a randomized controlled trial

**DOI:** 10.3389/fpubh.2024.1408026

**Published:** 2024-06-11

**Authors:** Janine Bacher, Christina Stadler, Eva Unternaehrer, Donja Brunner

**Affiliations:** Child and Adolescent Psychiatric Research Department, University Psychiatric Clinics, University of Basel, Basel, Switzerland

**Keywords:** cultural adaptation, effectiveness, randomized controlled trial, mental health, refugees, study protocol, adolescents

## Abstract

**Background:**

Adolescent refugees are particularly vulnerable to mental health problems, as they experience many risk factors associated with their resettlement at crucial stages of their physical and emotional development. However, despite having a greater healthcare needs than others, they face significant barriers to accessing healthcare services. Therefore, this study aims to test the effectiveness of a low-threshold, culturally adapted version of the skills training START NOW – START NOW Adapted - in reducing mental health problems among adolescent refugees.

**Methods:**

We will recruit 80 adolescent refugees (15–18 years) with symptoms of anxiety and depression or high perceived stress in Northwestern Switzerland. They will be randomly assigned to one of two study groups: an intervention group, receiving START NOW Adapted, and a control group, receiving treatment as usual (TAU). The intervention will last 10 weeks and will consist of one-hour sessions per week provided by a trained facilitator with the same cultural background, in the respective language. Assessments to collect depressive and anxious symptoms, perceived stress, social-ecological resilience, and emotion recognition abilities will be conducted pre-intervention, post-intervention (11 weeks later) and at the 3-month follow-up. Multilevel models will be computed with primary and secondary outcome measures as dependent variables. An effect of at least moderate size will be considered clinically relevant.

**Discussion:**

This randomized controlled trial aims to investigate the effectiveness of a culturally adapted version of START NOW, providing valuable insights to improve current health promotion for adolescent refugees in Switzerland (or rather lack thereof). Ultimately, the effects of START NOW may facilitate integration and promote healthy development while decreasing costs associated with treating migration- or conflict-related trauma.

**Clinical trial registration**: ClinicalTrials.gov, identifier: NCT06324864.

## 1 Background

Over the last few decades, Europe has experienced one of the largest increases in international migration, which has contributed to making migration a major social, political and public health challenge (1). The increased migration rates result from a combination of natural and man-made disasters, including social, economic, and political instability (1). As these disasters cannot be expected to decrease in the future, it is essential to establish optimal healthcare for migrants, especially refugees (2).

Refugees are a subset of migrants who differ in the reasons for their displacement, namely a well-founded fear of persecution, conflict, violence or other forms of danger (3). Children and adolescents below 18 years of age make up about half of the worldwide refugee population (4). Due to several risk factors for their psychological well-being, refugees have higher prevalence rates of depression, PTSD and other anxiety disorders compared to the host-country population (5, 6). Several systematic reviews have found prevalence rates of up to 40% for self-reported depression, 37% for self-reported PTSD, and 42% for self-reported anxiety (7, 8). Adolescent refugees are particularly vulnerable to mental health problems because they experience many of the risk factors associated with their resettlement at crucial stages of their physical and emotional development (9).

Risk factors for adolescent refugees’ psychological well-being can be categorized into pre-, peri- and post-migration stressors (10). They can also be conceptualized at different levels according to Bronfenbrenner’s socio-ecological perspective, whereby youth development takes place in a complex interplay of different levels (e.g., individual, family, school, community, and society) (11). At the individual level, exposure to traumatic events before migration is associated with higher levels of depression, PTSD, and anxiety, especially if these traumatic events involve severe interpersonal violence (12). Pre-migration trauma even persists to impact refugees’ mental health years after displacement (5). At the family level, refugees who experience separation from family members peri-migration show higher levels of PTSD and emotional problems (12). Post-migration, decreased family functioning correlates with more internalizing and externalizing issues (13). At the community level, lower social support post-migration increases the risk of depression, PTSD, and anxiety (14). At the society level, experiences of discrimination as well as staying in settings with lower supervision post-migration predicts symptoms of depression, PTSD, and anxiety (12).

However, despite having greater healthcare needs than others, adolescent refugees encounter significant barriers to accessing healthcare services (15, 16). Systemic barriers include the lack of healthcare services and long waiting lists (16). Even if refugees are given a place in the healthcare system, a financial barrier is the affordability thereof, including payment for treatment, transport and medication (17). Understanding the complex healthcare system and the lack of information about it is also a barrier for many refugees (15). Another barrier, particularly among young asylum seekers, is mistrust of the healthcare system and the fear that health information could play a role in the asylum procedure (15). Refugees also often face challenges due to language difficulties and the scarcity of accessible interpreters who are both professional and culturally sensitive (15, 16). To overcome these barriers, peer approaches would be necessary, in which a certain basis of trust and understanding already exists due to a common cultural background. These peers could interact with refugees in a manner that respects cultural norms and values, aiming to support their healing and resilience (18).

## 2 Objectives

To date, there is a significant lack of randomized controlled trials investigating the effectiveness of culturally adapted interventions for adolescent refugees provided by a trained facilitator with the same cultural background (19). Therefore, the primary aim of this RCT is to evaluate the effectiveness of a culturally adapted version of the group training START NOW in reducing mental health problems of adolescent refugees.

### 2.1 Endpoints

Symptoms of depression and anxiety will be the primary endpoints because of their high self-reported prevalence of up to 42% in refugee populations and therefore their high relevance to participants (7, 8). The intervention START NOW also aims to improve emotion regulation, mindfulness and interpersonal effectiveness, which are effective skills in decreasing symptoms of depression and anxiety (20–22).

Secondary endpoints include perceived stress, social-ecological resilience, and facial emotion recognition.

We chose perceived stress as a secondary endpoint because of the many pre-, peri- and post-migration stressors that are risk factors for refugees’ mental health. These risk factors could be better managed since the intervention START NOW aims at improving distress tolerance (22). Adding to that, perceived stress has been found to mediate effects of protective resilience factors such as self-esteem on depression (23).

We included social-ecological resilience as another secondary endpoint because it has often been used to assess the effectiveness of interventions in pre-post and longitudinal designs (e.g., (24)).

We chose the secondary endpoint of facial emotion recognition (ER) because performance on measures of emotion recognition is a first step in emotion regulation (25). Consistent with the content and goals of CBTs, certain types of biases in ER that are associated with depressive symptoms, such as labeling emotionally neutral stimuli as negatively valenced, can be reduced following the START NOW intervention (26, 27).

### 2.2 Hypotheses

Based on our primary and secondary endpoints, we have formulated the following hypotheses.

1. START NOW Adapted will be more effective than TAU in reducing symptoms of depression and anxiety, as assessed by the Hopkins Symptom Checklist (HSCL-25).
2. START NOW Adapted will be more effective than TAU in reducing perceived stress, as assessed by the Perceived Stress Scale (PSS-10).
3. START NOW Adapted will be more effective than TAU in increasing social-ecological resilience, as assessed by the Child and Youth Resilience Measure (CYRM-R).
4. START NOW Adapted will be more effective than TAU in increasing facial emotion recognition, as assessed by the Penn Emotion Recognition Task (ER-40).

Additionally, besides investigating the intervention’s effectiveness, we aim to examine potential mediators to uncover the mechanisms of action (28). Specifically, we hypothesize:

1. The relationship between the group condition and changes in depressive and anxious symptoms (HSCL-25) is mediated by stress reduction (PSS-10).
2. The relationship between the group condition and changes in depressive and anxious symptoms (HSCL-25) is mediated by increased resilience (CYRM-R).
3. The relationship between increased resilience (CYRM-R) and changes in depressive and anxious symptoms (HSCL-25) is mediated by stress reduction (PSS-10).

## 3 Methods

The protocol for this RCT was designed according to the Recommendations for Interventional Trials (SPIRIT; (29)) and was registered in the ClinicalTrials.gov trial database on March 21, 2024 (Trial registration number: NCT06324864). We will adhere to the guidelines outlined in the Consolidated Standards of Reporting Trials (CONSORT; (30)).

### 3.1 Study design

The study is a confirmatory, randomized controlled trial with two arms that takes place in a monocentric, national setting and assesses effectiveness.

To evaluate feasibility, qualitative semi-structured interviews will be conducted with specialists working with refugees prior to the intervention study. These interviews will follow an inductive and explorative approach and involve staff from institutions such as asylum centers and dormitories for unaccompanied minors. To estimate the feasibility and effectiveness of this trial, specialists will be interviewed about their daily challenges, language and cultural barriers, as well as potential difficulties with the implementation and adaptation of START NOW. The inclusion of qualitative information can help to better understand how a complex intervention such as START NOW can work and aid in the adaptation process of the intervention (31).

**Figure 1:**
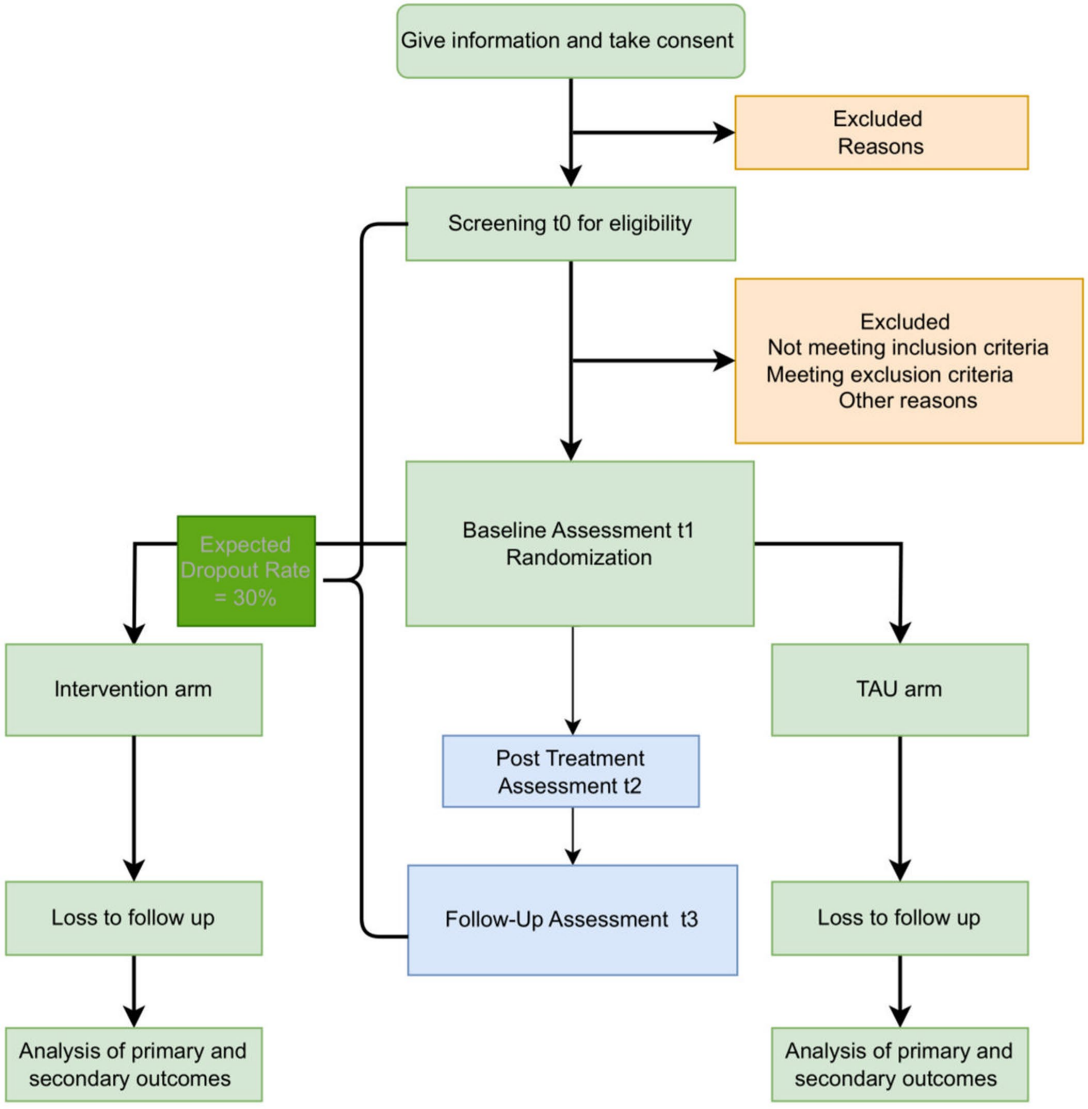
Study Flow

After the conduction of these interviews, possible participants taking part in the intervention study are screened for eligibility. For those who are eligible, the study duration will be 6 months, including three assessments (shown in Figure 1): a baseline assessment (t1), a post-treatment assessment (t2), and a 12-weeks follow-up assessment (t3). In case a participant has missed a session, they will be marked as absent and the number of sessions will be taken into consideration in the data analysis. To minimize bias, participants will be randomly assigned to one of two experimental group conditions, (1) group skills training using an adapted version of START NOW, (2) TAU. Randomization should take place within 2 weeks before the intervention, after the baseline assessment (t1) has been conducted. Between t1 and t2, the intervention phase takes place over a period of 10 weeks, with one session per week for each group. The study takes place in a group setting since conducting mental health interventions via group workshops, which aim to enhance social interaction was observed to have a positive impact on participants’ mental health (19). For ethical reasons and to increase study-related commitment, participants in the control group (receiving TAU) as well as non-eligible participants will be offered the chance to receive treatment after the study concludes.

### 3.2 Study procedure

The overall study duration will be 2 years, starting in March 2024 with recruitment and ending in March 2026. In collaboration with the Department of Economic, Social and Environmental Affairs of Basel-Stadt, the study team will recruit participants from social welfare, migration + integration and inpatient counseling facilities, such as residential homes for unaccompanied minors, as well as associated organizations such as the Center for Social Pedagogy and Psychotherapy in Basel. At this point, institutions will also be asked if some of their staff are willing to participate in qualitative semi-structured feasibility interviews before the start of the intervention study. In addition, we will contact associated networks for young people with a migration background, such as leisure activities or clubs and other institutions in Basel-Land. The relevant institutions and networks will be informed about the study by email or by telephone. Interested institutions will be given flyers and interested young people will be referred to our team.

On site or during a video call, the investigators (study team members) will explain to each participant the nature of the study, its purpose, the procedures involved, the expected duration, the potential risks and benefits, and any inconvenience that may be involved. Each participant will be informed that participation in the study is voluntary, that he or she may withdraw from the study at any time, and that withdrawal of consent will not affect subsequent medical care and treatment. If necessary, a translator (professional translator or bilingual native speaker from the institution staff) will also be present. All participants in the study will receive a participant information sheet and a consent form describing the study and providing sufficient information in the native language of the participants so they can make an informed decision about their participation in the study. The participant will be given 1 week to decide whether to participate. The formal consent of a participant, using the approved consent form, will be obtained before the participant is submitted to any study procedure. The consent form will be signed by the participant, sent with a prepaid envelope and dated upon arrival by the investigator or his designee. The date of receipt of the consent form is the date of inclusion for participation in the study. A copy of the signed informed consent will be given to the study participant. The consent form will be retained as part of the study records. The informed consent process will be documented and any discrepancies in the process described in the protocol will be explained.

After informed consent has been given, participants will be screened for eligibility. Any participants can withdraw from the study at any time without personal disadvantages and without having to give a reason. The applicants can also decide about termination of treatment in case of protocol violations or if the applicants believe continuation in the study would be detrimental to the participants` well-being. If participants withdraw their participation prior to randomization, other eligible individuals can move up. Premature withdrawals in the group training will not be replaced after the beginning of the intervention. Relevant additional treatments (e.g., individual psychotherapy) administered to the participant upon entry to the trial or at any time during trial participation are regarded as concomitant treatments and will be documented as well as concomitant medication.

After the screening process, eligible persons will be notified by the project management through their institution about their participation opportunity. If someone is not eligible, both the institution as well as the individual will be notified online via mail. If eligible participants still wish to take part, they are randomly assigned to a group. In general, randomization will be done within each institution by randomly assigning participants to either the intervention or control group using computer algorithms. If not enough adolescents within an institution wish to participate, we will make an adjustment to the general randomization process by creating cross-institutional groups. Participants will be randomized in separate steps for each gender group. In this way, we ensure a random distribution between the intervention and control groups within each gender group and a safe environment for all participants. This is in tune with cultural norms and expectations of the target population, and this way, our results are more likely to provide important insights around cultural idioms of distress that may be gender specific (32).

### 3.3 Eligibility criteria

The study population consists of adolescent refugees between the ages of 15 and 18 who are temporarily or permanently resettled in Switzerland and have given informed consent. In November 2023, the majority of new arrivals to the asylum procedure in Switzerland were from the following countries: Ukraine, Turkey, Afghanistan and Algeria (33). For this reason, our participants will presumably consist of homogeneous groups from these nations, as groups with the same migration background show greater intervention effectiveness than groups with different migration backgrounds (34).

The design of our study has been developed considering the conditions of regular clinical practice and focusing on outcomes crucial for making clinical decisions. In this context, the eligibility criteria enable the source population to represent diverse characteristics of external populations, accounting for comorbidities and varying compliance rates (35).

According to this study population, study inclusion criteria include:

- Being a refugee resettled in Switzerland temporarily or permanently
- Aged between 15 and 18
- Able to speak, read and understand one of the following languages: German, English, Turkish, Persian, Ukrainian, Arabic
- Able to give informed consent as documented by signature
- At least sub-clinical depressive symptoms assessed by the Hopkins Symptom Checklist-25 (HSCL-25); total score > 1.75 (36), or
- Having elevated levels of psychological distress assessed by Kessler Psychological Distress Scale (K10); total score > 20 (37)

Study exclusion criteria include:

- Suicidality assessed by the Suicide Behaviors Questionnaire-Revised (SBQ-R); total score > 6 (38)
- Planning to leave Switzerland in the next 6 months
- Concurrent CBT-based skills training similar to START NOW

We included underage participants since there is a lack of culturally adapted, low-threshold interventions targeting this demographic, notwithstanding the fact that approximately 50 % of all psychiatric disorders manifest in individuals at the age of 14 (39). Symptoms showing that a participant is unwilling to participate in the study will result in the participant being excluded from participation.

### 3.4 Interventions

#### 3.4.1 START NOW adapted

The intended low-threshold intervention START NOW promotes resilience, which refers to good developmental outcomes despite exposure to significant adversity (40). The existing START NOW training includes a manual with detailed instructions for group facilitation and a workbook for participants (22). The sessions consist of exercises and discussions wherein participants will learn skills and tools and will have the opportunity to practice, ask questions, and discuss the topics and approaches. The program combines aspects of cognitive behavioral therapy (CBT), motivational interviewing (MI), dialectical behavior therapy (DBT), acceptance and commitment therapy (ACT) and trauma-sensitive care (22). Studies in different contexts have shown START NOW’s effectiveness in terms of reducing oppositional and aggressive symptoms (41), reducing hospitalization rates (42), improving mental health functioning (43) and increasing satisfaction rates (44). There is a need to culturally adapt this existing intervention, which has previously been used with Western populations, since effective psychological interventions are rooted in a cultural context and must be consistent with clients’ cultural beliefs (45). Moral and ethical obligations therefore dictate that mental health practitioners take into account cultural contexts and values relevant to the well-being of clients (46). The same applies to the development of interventions, as cultural adaptation positively affects the outcome of treatment and has shown to be more effective compared to conventional Western, bona-fide interventions (47, 48). Effective interventions can use culturally sensitive approaches that avoid pathologizing refugees’ experiences and instead honor cultural systems and values to promote recovery and resilience processes (18).

Meta-analyses have shown that the effectiveness of adapted interventions increases with the number of adaptation elements implemented (49). According to Kananian et al.’ (50) proposal of deep structural adaptations used in the development of Culturally Adapted Cognitive Behavioral Therapy (CA-CBT) for PTSD (51), different elements can be divided into specific and unspecific components, surface adaptations, mode of delivery, translation, and adaptation of materials. Specific components include adapting session content to the refugee experience and using positive imagery to overcome cultural barriers (52). Unspecific components refer to cultural concepts of distress, including explanatory models (i.e., aetiological assumptions) and idioms of distress (i.e., the expression of symptoms) (53).

Regarding specific components in START NOW Adapted, we will adapt both the workbook for participants as well as the manual for facilitators by including exemplary experiences of young refugees instead of the original ones. In order to capture these experiences, we will gain insights from social workers who work directly with young refugees. It is important that the facilitator conveys that these experiences are exemplary and that everyone has individual but possibly similar experiences. Positive images, such as different weather conditions or a color palette drawn to describe the current emotional state, serve as an easier introduction to the topics. With regards to unspecific components, previous research has shown that emotions are understood differently in different cultures (53). Therefore, explanations in the workbook and manual will be kept to a minimum. The facilitators are instructed to ask about the individual understandings in their groups and to include the young people’s explanations in the training.

Superficially, the comics and characters will be adapted to represent more diverse cultures and better opportunities for identification.

Regarding the mode of delivery, group-based interventions have shown to be a successful method for mental health intervention among refugee populations (19). Therefore, START NOW Adapted will be led by 10 group discussions with a manual guiding the facilitators through the sessions. The facilitator is a trained individual from the same cultural background and supervised by a START NOW member of the research team.

All materials will be translated into German, English, Turkish, Persian, Ukrainian and Arabic.

In the introduction, participants learn about emotions and are introduced to the START NOW skills. These skills, called SLOW DOWN, TAKE A STEP BACK, ACCEPT, RESPECT and TAKE ACTION, are strategies or techniques that can be used to better manage stress and difficult emotions. Participants will also get to know six fictional teenagers who are often stressed and get into trouble because they cannot regulate their emotions. During the sessions, the teenagers will be used as examples to illustrate situations.

In the second session, participants are introduced to the ABC model of rational-emotional behavior therapy (Adverse Event - Beliefs - Consequences) and learn that every feeling has an important purpose. In the third session, participants learn about the importance of mindfulness and do a mindful breathing exercise (SLOW DOWN). In the fourth session, participants are invited to step back from negative thoughts. They learn to observe them and to identify errors in thinking (TAKE A STEP BACK). In the fifth session, they learn to accept their negative feelings instead of fighting them, so that they do not get in the way of their goals. Participants are also guided to define goals and values that are important to them (ACCEPT). In the sixth session, participants learn that respect for themselves is an important prerequisite for recognizing when their own needs are being violated, and that respect for others is important for having good relationships and friendships. They check themselves for unequal relationship patterns and are given tips on how to set their own limits and not overstep them with others (RESPECT). In the seventh session, participants are taught to take action to achieve their goals by identifying obstacles and dividing them into things they can change and things they cannot (TAKE ACTION). In the eighth session, participants are given additional skills to regulate their emotions by not letting them reach dangerous levels and by observing their early warning signals.

Sessions 9 and 10 explicitly target anxiety and depression. Participants learn which unhelpful strategies they might use to cope with anxiety and how to manage anxiety using the START NOW strategies they have learned in the previous sessions. In the last session on depression, mechanisms are described to conquer depressive feelings, for example by acting in the opposite way to how they feel at that moment or identifying thought errors.

#### 3.4.2 Treatment as usual

The treatment as usual (TAU) group will receive their usual treatment. We will evaluate any potential modifications in treatment as usual for participants throughout the study, which will be considered during the statistical analysis or possibly lead to the exclusion of the case. Following the conclusion of the study, participants in the TAU group will have the option to receive the intervention START NOW Adapted for ethical reasons and to increase study-related commitment.

### 3.5 Study measures

An overview of all study measures can be found in Table 1. All questionnaires will be employed online via REDcap. They will be used in German, English, Turkish, Persian, Ukrainian and Arabic. Questionnaires that are not yet available in these languages will be translated using the Multiple-Forward-Approach.

**Table 1:** Assessment summary.

	Screening (t0)	Baseline (t1)	Post-intervention (t2)	Follow-up (t3)
Sociodemographic data	x			
Screening instruments				
K-10	x			
SBQ-R	x			
WHO-5	x			
Primary outcome				
HSCL-25	x	x	x	x
Secondary outcomes				
PSS-10		x	x	x
CYRM-R		x	x	x
ER-40		x	x	x
Other measures				
RPMS		x		
LEC-5		x		
CSQ-8			x	x

The table shows a summary of all study assessments.

#### 3.5.1 Screening measures

Screening measures include demographics such as age or country of origin, inclusion criteria including the HSCL-25 and the K10, exclusion criteria including the SBQ-R, and the World Health Organization Well-Being Index (WHO-5).

The K10 is a 10-item self-report questionnaire aimed at assessing overall distress levels. It gathers information on anxiety and depressive symptoms experienced by an individual within the past 4 weeks. Participants answer on a 5-point Likert Scale (1 = none of the time, 5 = all of the time), with scores ranging from 10 to 50. Higher scores indicate a higher possibility that participants suffer from a mental disorder.

The SBQ-R is a brief, 4-item self-report measure of past suicidal behavior. The questionnaire is used with adolescents between the ages of 13 and 18. Each question is rated on its own scale, with specific point values assigned to the answers and the total score ranging from 3 to 18 points. A higher score indicates a greater likelihood of engaging in suicidal behavior.

The WHO-5 is a brief self-report assessment used to measure mental well-being during the past 2 weeks. It includes five items with positive wording, each rated on a 6-point Likert scale (0 = at no time, 5 = all of the time). The total raw score, which ranges from 0 to 25, is multiplied by 4 to generate the final score. A high score indicates a higher level of well-being.

#### 3.5.2 Primary outcome

The primary outcome, symptoms of depression and anxiety, will be measured by the HSCL-25 (36) at baseline (t1), at the end of the intervention (t2) and 12 weeks after the end of the intervention (t3) to determine the effectiveness of the intervention. The HSCL-25 is a 25-item self-report questionnaire, on which participants rate if physical, emotional, or psychological symptoms indicating depression, anxiety, and trauma have affected them over the past week. Answers are given on a 4-point Likert Scale (1 = not at all; 4 = extremely), and total scores can range from 25 to 100, with higher scores indicating greater levels of depression, anxiety and trauma, respectively.

#### 3.5.3 Secondary outcomes

The secondary objective perceived stress will be measured by the PSS-10 (54) at baseline (t1), at the end of the intervention (t2) and 12 weeks after the end of the intervention (t3). The PSS-10 is a 10-item self-report questionnaire including two subscales, on which participants report life as unpredictable, uncontrollable, and overloading over the previous month. Answers are given on a 5-point Likert Scale (0 = never, 4 = very often). Higher scores indicate higher levels of perceived stress.

The secondary objective social-ecological resilience will be measured by the CYRM-R (55) at baseline (t1), at the end of the intervention (t2) and 12 weeks after the end of the intervention (t3). The CYRM-R is a 17-item self-report questionnaire measuring resilience that has been validated in 11 different countries (55). Items include social integration and support, autonomy and fair treatment and can be answered on a 3- point Likert Scale (1 = no, 3 = yes). Higher scores indicate more available protective factors and higher social-ecological resilience.

The secondary objective facial emotion recognition will be measured using the ER-40 (56) at baseline (t1), at the end of the intervention (t2) and 12 weeks after the end of the intervention (t3). The ER-40 consists of 40 color photographs of static multicultural faces expressing four basic emotions (i.e., happiness, sadness, anger or fear) and neutral expressions. Participants quickly select the appropriate emotion label for each face. They then rate their confidence in the accuracy of their response on a scale from 0 (not at all confident) to 100 (extremely confident). Accuracy scores range from 0 to 40. The ER-40 has strong psychometric properties and is recommended for use in clinical trials (57).

#### 3.5.4 Other measures

The Refugee Post-Migration Stress Scale (RPMS) (58) is a self-report questionnaire consisting of 21 items that assess various stressors experienced by refugees after migrating to a new country and will be measured at baseline (t1). The scale was originally developed for migration to Sweden and adapted to the Swiss context. Participants rate how often they have experienced each stressor after resettlement in Switzerland on a 5-point Likert Scale (1 = never; 5 = always). Total scores on the RPMS can range from 21 to 105, with higher scores indicating higher levels of post-migration stress.

The Life Events Checklist for DSM-5 (LEC-5) will be employed at baseline (t1) as a self-report questionnaire used to assess exposure to events that could potentially cause post-traumatic stress disorder (PTSD), depression, or distress. Participants indicate varying levels of exposure to each type of potentially traumatic event included on a 6-point Likert Scale (1 = does not apply, 6 = happened to me). The LEC-5 does not provide a total or composite score.

To assess the strengths and weaknesses of the intervention, participants will fill out the Client Satisfaction Questionnaire (CSQ-8) at the end of the intervention (t2). The CSQ-8 is a self-report questionnaire used to assess client satisfaction with services received. It consists of 8 items that measure various aspects of satisfaction, such as the perceived helpfulness of the service, the extent to which the client’s needs were met, and overall satisfaction with the care received. Each item is rated on a 4-point Likert Scale.

## 4 Statistics

### 4.1 Sample size

The sample size is estimated based on a power analysis for repeated measures ANOVA including within-between interaction with 3 measuring time-points (t1, t2 and t3) and 2*2 groups (intervention vs. TAU, female vs. male). Standard parameters are set at alpha = 0.05, power = 0.95, and a clinically meaningful medium effect size *f* = 0.25. Considering an attrition rate of 30% (59), the total number of participants should be 80, with approximately 40 participants in the treatment (treatment groups of 5 to 10 participants each) and 40 participants in the control group.

### 4.2 Statistical analyses

For statistical analyses, we will use R with its graphical user interface R Studio. The significance level will be 0.05 for all analyses. An effect of at least moderate size will be considered as clinically relevant. To test our hypotheses, we will use multilevel models with primary and secondary outcome measures as dependent variables (three individual models for depression, perceived stress, and emotion recognition), and with timepoint (baseline, post-treatment, follow-up) and intervention condition (START NOW vs. TAU) including their interaction as independent variables. A significant interaction between intervention condition and time suggests a difference in symptom change depending on treatment group. In case of significant main or interaction effects, we will evaluate post-hoc contrast considering time and intervention condition. Treatment group will be added as level two in the multilevel framework.

Using multilevel models will allow to analyze data on the observational level, which provides more information in contrast to calculating change scores or using repeated measures ANOVA, increasing measurement accuracy and power. In addition, multilevel mixed models can handle missing observations and allow to examine random effects at the intercept and slope level. Furthermore, we will test model improvement when considering quadratic versus linear trends for time, random effects for time, and correlation structure between timepoints. Covariates, including participant age, gender, and post-migration stress will be added if they differ between intervention groups. On group level, possible covariates include facilitator, institution or cultural background.

To analyze perceived stress and resilience as potential mechanisms in treatment success, we will use bias-corrected bootstrap mediation models with intervention condition as predictor, change in symptoms of depression as outcome, and change in stress/resilience as mediator. Change in symptoms of depression and indicators of stress/resilience will consist of participant’s random slope for time extracted from the multilevel mixed models depending on results of the first hypothesis.

The qualitative interviews will apply an inductive approach and follow empirical guidelines as suggested by Mayring (60). The aim is to conduct an estimate of five to eight interviews across different institutions, each of around 30 min in length. After recording (audio), the interviews will be transcribed and the audio files will be destroyed. Pertaining to the inductive categorization, evaluation and illustration of the qualitative data, MAXQDA will be employed (61).

## 5 Discussion

### 5.1 General discussion

This study protocol presents a randomized controlled trial testing the effectiveness of a culturally adapted version of START NOW in reducing mental health problems in refugees. To date, there is a significant lack of RCTs investigating the effectiveness of culturally adapted interventions for adolescent refugees (19). Consequently, the present study considers the need to create an intervention method that combines different theoretical rationales while addressing core problems of adolescent refugees and applying culturally adapted strategies and materials. Our population of interest addresses the fact that ethnic minorities are generally underrepresented in clinical trials in high-income countries (62).

The strengths of this study also include the highly ecological validity as it measures the extent of beneficial effects within real-world conditions (63). Our study design also adapts to the need of implementing and evaluating interventions within the community where clients reside (64). This is of major relevance for staff workers dealing with adolescent refugees since it saves time and cost. Thus, the implementation of evidence-based interventions within institutions is needed to strengthen the chance of continuous care and diminish the stigma associated with mental health services (65). Therefore, the findings of this study will be highly relevant to refugee health care and are intended to stimulate further discussion on how to improve, if not directly increase, the quality of care for adolescent refugees.

### 5.2 Limitations

One of the challenges of this project may be self-selection bias if certain refugees are more likely to volunteer for the intervention. This could affect the generalizability of the results to the wider refugee population. To reduce this bias, we offer high, but still appropriate, incentives of 20 CHF in vouchers for each assessment (t1 – t3). There also may be differences in the delivery of the intervention or usual care between facilitators. To counteract this bias, all facilitators receive extensive pre-training that provides the same content to all. Additionally, facilitators will be included as possible influences at level two in the multilevel framework.

Another limitation is the lack of blinding among participants and facilitators, a frequent cause of bias in RCTs that include non-pharmacological interventions. Known problems associated with lack of patient blinding, such as response bias with self-reported outcomes, relate primarily to studies with a predominantly explanatory aim (66). For instance, attrition rates may be simultaneously regarded as both sources of bias and outcomes influenced by the intervention (66). It is therefore important to understand and address the reasons for dropout. In multilevel models, a sub-model accounts for missing data. The selection model improves the analysis of growth curves by using logistic regressions to show how likely it is to have missing data at each time. Meanwhile, the pattern mixture approach looks at each group of missing data separately and then combines the results (67). Thereby, multilevel modeling makes use of all available information without needing to resort to list wise deletion. Another method of minimizing bias is that only well-validated questionnaires will be used during assessments.

### 5.3 (Forecast) expected execution dates

Promotion and initial recruitment of participants: March 2024.

First baseline assessment: August 2024.

Last follow-up assessment: March 2026.

Publication of results: December 2026.

## 6 Conclusion

Overall, results of this study will help inform about the feasibility and effects of alternative health promotion in an underserved population (i.e., refugees) characterized by a lack of health services and continuity of care. Insights may help improve current health promotion of adolescent refugees in Switzerland (or rather lack thereof) through providing a feasible skills training equipped to overcome the barriers to accessing adequate care services. Ultimately, effects of START NOW Adapted on depression may facilitate positive life outcomes and decrease costs associated with treating migration- or conflict-related trauma. If these effects prove to be clinically relevant in this RCT, the goal is to make START NOW Adapted widely available to other refugee populations in the future.

## Ethics statement

The studies involving humans were approved by Ethics Committee Northwestern and Central Switzerland (EKNZ). The studies were conducted in accordance with the local legislation and institutional requirements. Written informed consent for participation was not required from the participants or the participants’ legal guardians/next of kin because participants but not their legal guardians are required to give their informed written consent. In Switzerland, clinical trials presenting low risk (risk category A) require informed written consent only from participants once they are above 14 years.

## Author contributions

JB: Conceptualization, Data curation, Formal analysis, Investigation, Methodology, Project administration, Visualization, Writing – original draft, Writing – review & editing. CS: Conceptualization, Funding acquisition, Resources, Writing – review & editing. EU: Formal analysis, Methodology, Writing – review & editing. DB: Conceptualization, Data curation, Funding acquisition, Investigation, Project administration, Supervision, Writing – review & editing.
